# ^68^Ga-FAPI and ^18^F-FAPI PET/CT for detection of nodal metastases prior radical cystectomy in high-risk urothelial carcinoma patients

**DOI:** 10.1007/s00259-025-07239-6

**Published:** 2025-04-24

**Authors:** Lena M. Unterrainer, Hans P. Schmid, Sophie C. Kunte, Adrien Holzgreve, Johannes Toms, Paula Menold, Clemens C. Cyran, Alexander Karl, Stephan Tschirdewahn, Stephan T. Ledderose, Lennert Eismann, Alexander J. Tamalunas, Maximilian Scheifele, Christian G. Stief, Marcus Unterrainer, Jozefina Casuscelli, Gerald B. Schulz

**Affiliations:** 1 Department of Nuclear Medicine, LMU University Hospital, LMU Munich, Marchioninistrasse 15, 81377 Munich, Germany; 2 Bavarian Cancer Research Center (BZKF), Partner Site Munich, Munich, Germany; 3 Ahmanson Translational Theranostics Division, Department of Molecular and Medical Pharmacology, David Geffen School of Medicine, University of California los Angeles, Los Angeles, CA USA; 4 Department of Urology, Krankenhaus Barmherzige Brüder, Munich, Germany; 5 Department of Radiology, LMU University Hospital, LMU Munich, Munich, Germany; 6 Department of Urology, Klinikum Kempten, Kempten, Germany; 7 Institute of Pathology, LMU Munich, Munich, Germany; 8 Department of Urology, LMU University Hospital, LMU Munich, Munich, Germany; 9 Die RADIOLOGIE, Munich, Germany

**Keywords:** FAPI PET, F-18 FAPI, Ga-68 FAPI, Urothelial carcinoma

## Abstract

**Introduction:**

To determine the best therapeutic strategy for muscle-invasive bladder cancer (BC), the accuracy of lymph node staging is of paramount importance. However, diagnostic performance of conventional computed tomography in BC prior to radical cystectomy (RC) remains unsatisfactory. There is an increased interest in evaluating ^18^F-FAPI PET/CT for hybrid imaging due to their logistical advantages compared to [^68^Ga]Ga-based FAPI tracers in clinical routine. Recently, the potential diagnostic value of [^68^Ga]Ga-FAPI- 46 PET/CT was demonstrated in BC. Thus, we aimed to examine the diagnostic performance of [^18^F]F-FAPI- 74 and [^68^Ga]Ga-FAPI- 46 PET/CT for preoperative evaluation of locoregional lymph node metastases.

**Methods:**

Fifty-one patients underwent FAPI PET/CT with either [^68^Ga]Ga-FAPI- 46 (*n* = 23) or [^18^F]F-FAPI- 74 (*n* = 28) prior to RC and PLND. SUV_max_, SUV_mean_ and the ratio between the SUV_max_ of lymph nodes and the SUV_mean_ of the background (SUV_max_lymph node_/SUV_mean___background_) were assessed. Additionally, short axis diameter (SAD) for a representative lymph node were documented in each lymph node region (*n* = 123) and compared to histopathological findings. Each scan was interpreted visually and quantitatively. ROC-analyses were performed to determine cut-off values with highest diagnostic accuracy.

**Results:**

20/123 (16.3%) lymph node regions showed UC lymph node metastases. Histopathologically positive lymph nodes were associated with a significantly higher FAPI uptake compared to negative lymph nodes regarding SUV_max_, SUV_mean_ values and SUV_max_lymph node_/SUV_mean___background_ ratios. Visual analysis based on FAPI uptake showed a sensitivity and specificity, PPV and NPV of 63.6%, 95.8%, 77.7%, and 92.0% for [^68^Ga]Ga-FAPI- 46 and 55.5%, 98.1%, 83.3%, and 93.1% for [^18^F]F-FAPI- 74, respectively. ROC analysis revealed an optimal cut-off for SUV_max_, SUV_mean_ and SUV_max_lymph node_/SUV_mean___background_ of 1.35, 1.20 and 5.95 for [^68^Ga]Ga-FAPI- 46 and 1.55, 1.25 and 4.15 for [^18^F]F-FAPI- 74 to discriminate between histopathologically proven lymph node metastases and non-malignant lymph nodes resulting for example using SUV_max_ in a sensitivity and specificity, PPV and NPV of 81.8%, 89.5%, 64.2%, 95.5% for [^68^Ga]Ga-FAPI- 46 and 100%, 81.8%, 47.3%, 100% for [^18^F]F-FAPI- 74, respectively. CT visual analysis of locoregional lymph nodes showed a sensitivity, specificity, PPV and NPV of 30.0%, 97.0%, 66.6% and 87.7%, respectively. ROC analysis regarding SAD revealed a cutoff at 0.8 cm with a sensitivity, specificity, PPV and NPV of 75.0%, 84.4%, 48.3%, 94.5%, respectively.

**Conclusion:**

Overall, FAPI PET imaging shows a significantly higher sensitivity than CT analysis for detection of locoregional lymph node metastases in UC. [^18^F]F-FAPI- 74 demonstrates a comparable diagnostic performance compared to [^68^Ga]Ga-FAPI- 46. Of note, the quantitative analysis with a pre-defined SUV_max_ as well as SUV_mean_ values, and SUV_max_lymph node_/SUV_mean___background_ ratio-based cut-offs provided a higher sensitivity compared to visual assessment.

**Supplementary Information:**

The online version contains supplementary material available at 10.1007/s00259-025-07239-6.

## Introduction

The diagnosis and treatment of muscle-invasive carcinoma of the bladder (MIBC) represents an interdisciplinary challenge. Current guidelines recommend radical cystectomy (RC) with pelvic lymph node dissection (PLND) in combination with neoadjuvant therapy for high-risk, fit patients with T2-T4a, cN0-Nx, cM0 stage [1, 2]. On the one hand, PLND serves to identify micro metastases in inconspicuous lymph nodes and therefore can lead to clinically relevant upstaging and the indication for adjuvant treatments; on the other hand, it can reduce the tumor burden. Thus, node-negative patients can also benefit from PLND [3, 4]. The presence of lymph node metastases is decisive for the outcome of patients and is associated with a lower recurrence free survival (RFS) [5, 6]. Studies suggest them as the most powerful predictor of cancer-specific survival [7]. Therefore, in addition to determining the local tumor extension, one of the most crucial objectives of preoperative non-invasive diagnostics is to identify lymph node metastases. This helps to identify which patients would benefit from RC and who should receive systemic therapy instead. Conventional imaging, such as CT and MRI, have low sensitivity and specificity in the detection of lymph node metastases, with a potential for up to 40% of false negative findings [1, 2, 8–11]. In particular, smaller metastases in morphologically unremarkable lymph nodes frequently go undetected in CT/MRI imaging [12]. The assessment of lymph node size for the purpose of identifying metastatic lesions can result in a significant reduction in sensitivity (45.5%) and specificity (91.5%) [13]. Even though the use of ^18^F-FDG-PET/CT for routine clinical staging has been investigated, it is not recommended by current guidelines [1, 2].

Fibroblast activation protein inhibitor (FAPI) PET/CT is a novel approach to oncological diagnostics. The target structure is the fibroblast activation protein (FAP), a membrane-bound peptidase, which is increasingly expressed in cancer activated fibroblasts (CAFs) in the stroma of tumor cells. These are particularly present in solid tumors with a poor prognosis and are associated with tumor progression and immunosuppression [14]. Furthermore, evidence indicates a correlation between FAP expression and tumor aggressiveness [15]. In a direct comparison of ^18^F-FDG and ^68^Ga-FAPI, a preoperative PET/CT staging study of lymph node metastases in bladder carcinoma demonstrated superior detection rates and tumor-to-background ratios (TBR) for FAP imaging [16]. We have previously demonstrated the efficacy of pre-therapeutic ^68^Ga-FAPI diagnostics [17, 18]. The reduced renal excretion and lower urinary activity of [^18^F] ligands in comparison to [^68^Ga] ligands provides improved diagnostic capabilities regarding small lesions in urogenital carcinomas [19]. Both tracers demonstrated comparable performance in identifying malignant lesions and TNM staging in prostate carcinoma [20]. They have further clinical advantages, including a longer half-life than ^68^Ga labelled radiotracers and the capacity to produce larger batches for medical supply [21].

The objective of this study was to investigate in greater detail the diagnostic value of [^68^Ga]Ga-FAPI- 46 and [^18^F]F-FAPI- 74 in high-risk urothelial carcinoma patients prior to cystectomy regarding the detection of locoregional lymph node metastases: We analyzed the correlation between preoperative FAPI imaging findings and the corresponding histopathological results of bladder tumor and lymph node specimens. The aim was a preoperative assessment of the locoregional lymph nodes to identify lymph node metastasis by elevated FAPI uptake and to compare both FAPI tracers in their performance regarding the detection of locoregional lymph node metastases in BC.

## Material and methods

### Patient characteristics

The study included 51 patients with histologically confirmed diagnosis of muscle-invasive urothelial carcinoma of the bladder (baseline characteristics in supplementary information). Inclusion criteria encompassed patients with histopathologically proven MIBC before RC and PLND. All patients received a preoperative whole body FAPI PET/CT at a mean 9.9 ± 13.6 days before cystectomy, including 23 patients with [^68^Ga]Ga-FAPI- 46 and 28 patients with [^18^F]F-FAPI- 74 for locoregional lymph node assessment and exclusion of distant metastases. Of the aforementioned patients, 43 patients underwent RC with PLND, while 8 patients did not undergo PLND. Written consent for imaging was obtained from all patients. This retrospective analysis was approved by the Ethics Committee of the LMU Munich as part of the registry study (# 24–0255) in accordance with the guidelines of the Declaration of Helsinki and its subsequent amendments.

### Radiosynthesis

[^68^Ga]Ga-FAPI- 46 and [^18^F]F-FAPI- 74 were manufactured by SOFIE (21,000 Atlantic Blvd., Ste 730, Dulles, VA). Radiosynthesis of [^68^Ga]Ga-FAPI- 46 was performed as previously described [17]. Following the regulations of the German Pharmaceuticals Act §13(2b), the production of [^18^F]F-FAPI- 74 was performed under the direct responsibility of the applying physician. The precursor FAPI- 74 was provided in GMP quality by SOFIE (Dulles, VA, USA).

[^18^F]Fluoride was produced on site using a 16.5 MeV PETtrace cyclotron (GE Healthcare, Uppsala, Sweden) via the ^18^O(p,n)^18^F nuclear reaction by proton bombardment of enriched [^18^O]H_2_O (Rotem Industries, Arava, Israel). The radiosynthesis of [^18^F]F-FAPI- 74 was performed on an AllinOne module (Trasis, Liège, Belgium) using a FAPI- 74 cassette and reagent kit (Trasis, Liège, Belgium). The radiosynthesis was prepared by preformulating aluminum chloride in dimethylsulfoxide and acetonitrile as well as pH adjustment to a value of 4 to 5 with ascorbic acid. [^18^F]Fluoride with starting activities of 47.2 ± 13.4 GBq was trapped on a QMA cartridge and eluted by a NaCl-based eluent. The radiolabeling step was performed at 70 °C for 10 min and the crude product was purified by SPE followed by formulation in a sodium ascorbate-containing matrix. The short sequence yielded radioactivity yields (RAY) of 63.0 ± 9.2% (*n* = 36) with a radiochemical purity (RCP) of 99.8 ± 0.1% (n = 36) without HPLC purification. The product was sterile filtered through a Merck Cathivex-GV and subsequently dispensed under clean room class A conditions. The specific activity of the final product at EOS was between 1729 GBq/µmol and 10,286 GBq/µmol. The final product underwent a quality control procedure in accordance with the local regulations.

## Imaging

### Acquisition

PET/CT was acquired 70.3 ± 15.1 min after tracer injection using a Siemens Biograph mCT flow or Biograph 64 PET/CT scanner (Siemens Healthineers, Erlangen, Germany). Mean injected activity was 208 ± 41 MBq. If no contraindication was given, patients received furosemide intravenously (Furosemid-ratiopharm 20 mg/2 mL injection solution, ratiopharm GmbH, Ulm, Germany). The PET component was acquired from skull base to mid-thigh with a full dose contrast-enhanced CT (mean tube voltage 108.3 ± 26.4 kV, mean tube current 182.2 ± 39.2 mAs, mean contrast dye volume 116 ± 18.0 ml) or, in case patients received recent previous diagnostic CT imaging (26.1 ± 12.6 days), a low dose CT for attenuation correction.

### Analysis

PET imaging was evaluated by two experienced nuclear medicine physicians using Hermes Hybrid Viewer software package (Hermes Medical Solutions, Stockholm, Sweden) followed by a consensus read in that the discordant cases were assessed. For background FAPI uptake, SUV_mean_ values were measured using a 2 cm volume of interest (VOI) sphere in healthy liver tissue, blood pool and mesenterial or retroperitoneal fat and a 1 cm VOI in renal cortex similar to previous biodistribution studies [16].

In each lymph node region, the lymph node with the highest SUV_max_ and its respective SUV_mean_ as well as short axis diameter (SAD) was evaluated as previously described [17]. To compare the lymph node uptake to the background, the ratio of SUV_max_ of the lymph node and SUV_mean_ of the mesenterial/retroperitoneal fat was calculated. In addition to that, we correlated the SAD to SUV_max_, SUV_mean_ and the SUV_max_lymph node_/SUV_mean___background_ ratio.

The lymph node regions were classified according to anatomical regions, encompassing common iliac, internal iliac, external iliac, and obturator or pelvic (each left/right, respectively). After PLND, lymph nodes were examined histopathologically for malignancy. In case of a metastatic finding in histopathology, the corresponding lymph node region was rated as positive lymph node region; without lymph node metastases in histopathology, the region was rated as negative lymph node region. Histopathological results were compared to the PET(/CT) and CT findings, which were then categorized as either positive or negative lymph nodes. PET positive lymph nodes were defined as lymph nodes with a visually markedly elevated uptake above the background. CT-positive lymph nodes were rated positive as previously described according to established classifications [22].

### Statistics

Statistical analysis was performed using Graph Pad Prism version 8 (GrapPadSoftware, Boston, MA). Continuous variables were specified as mean ± standard variation. Statistical significance was evaluated using non-paired t-test and the Wilcoxon test. Correlation was calculated using Pearson and Spearman correlation. Interrater reliability was calculated using Cohens kappa. To assess the optimal cutoff for histopathologically proven and non-proven lymph node metastases, a receiver-operating-characteristic (ROC) analysis was performed and the area under the curve was assessed. Youden J index was used to evaluate the best discriminative cut-off value. Statistical significance was determined with a *p*-value < 0.05.

## Results

### Patients

51 (45 men; 6 women) patients with high grade BC at a mean age of 72.5 ± 9.4 years were included, 8 patients did not undergo PLND. The majority of patients presented with Grade 3 MIBC (82.6%) and T2 (37.2%) and T3 (27.4%) stage. Preoperative FAPI PET/CTs were performed at a mean of 9.9 ± 13.6 days before surgery. All patients received local pretreatment with transurethral resection of the bladder, 9/51 patients (17.6%) underwent systemic therapy such as chemotherapy and immunotherapy prior to FAPI PET/CT imaging. Histopathologically confirmed lymph node metastases were found in 12/43 patients (27.9%).

### Imaging

In the included 43 patients with concomitant PLND during cystectomy, a total of 123 lymph node regions were examined: 20/123 (16.2%) showed histopathologically proven metastases, within these, 9/123 (7.3%) were in the [^18^F]F-FAPI- 74 group and 11/123 (8.9%) were in the [^68^Ga]Ga-FAPI- 46 group. In total, 538 lymph nodes were removed with a median of 12 lymph nodes per patient, 28/538 showed lymph node metastases (5.2%): 14/28 were metastases in the cohort of [^18^F]F-FAPI- 74 (positive LN ratio: 14/303, 4.6%) and [^68^Ga]Ga-FAPI- 46 (positive LN ratio: 14/235, 5.9%) patients.

### Detection of lymph node metastases

The mean SUV_max_ of all included lymph nodes for [^18^F]F-FAPI- 74 was 2.3 ± 2.8 and for [^68^Ga]Ga-FAPI- 46 4.3 ± 8.5. Mean SUV_mean_ for [^18^F]F-FAPI- 74 was 1.7 ± 1.9 and for [^68^Ga]Ga-FAPI- 46 3.9 ± 7.8. Overall mean SUV_max_lymph node_/SUV_mean___background_ ratio was 6.4 ± 11.3, for [^18^F]F-FAPI- 74 4.0 ± 4.4, for [^68^Ga]Ga-FAPI- 46 9.6 ± 15.9. Overall, the mean SAD was 0.7 ± 0.2 cm for both tracers, for [^18^F]F-FAPI- 74 0.7 ± 0.2 cm and for [^68^Ga]Ga-FAPI- 46 0.6 ± 0.2 cm.

Lymph nodes in a lymph node region with at least one lymph node metastasis according to histopathology, showed a mean SUV_max_ of 6.2 ± 4.4 and mean SUV_mean_ of 4.5 ± 3.3, mean SUV_max_lymph node_/SUV_mean___background_ ratio of 10.5 ± 7.2 for [^18^F]F-FAPI- 74 and SAD 1.1 ± 0.3 cm vs. mean SUV_max_ 8.9 ± 11.4, mean SUV_mean_ of 6.0 ± 5.5 and SUV_max_lymph node_/SUV_mean___background_ ratio of 20.6 ± 21.1 and SAD 0.8 ± 0.2 cm for [^68^Ga]Ga-FAPI- 46.

Lymph node regions without metastatic lymph nodes showed a mean SUV_max_ of 1.4 ± 0.9, mean SUV_mean_ of 1.1 ± 0.5, mean SUV_max_lymph node_/SUV_mean___background_ ratio of 2.5 ± 1.5 for [^18^F]F-FAPI- 74 and mean SAD 0.6 ± 0.2 cm vs. mean SUV_max_ 2.4 ± 6.2, mean SUV_mean_ of 1.8 ± 4.5, mean SUV_max_lymph node_/SUV_mean___background_ ratio of 5.0 ± 10.4 and mean SAD 0.6 ± 0.2 cm for [^68^Ga]Ga-FAPI- 46.

**Fig. 1:**
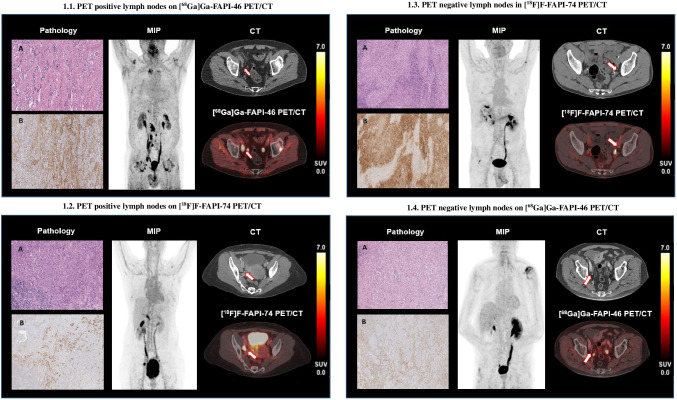
Different FAP expression patterns in lymph node metastases of urothelial carcinoma. **1.1.** PET positive lymph nodes on [^68^Ga]Ga-FAPI- 46 PET/CT, Tumor infiltrates in **A** (H&E) show a pronounced desmoplastic reaction with strong stromal FAP expression in immunohistochemistry (**B**, FAP-IHC) (obturator right, SUV_max_ 41, SUV_mean_ 19, SAD 1.1 cm). In the right obturatoric space FAPI PET/CT shows a lymph node with high FAP expression (arrow). Next to it physiologic exrection of the tracer in the ureter, same on the left side. Also visible physiological radiotracer accumulation in degenerative alterations of the adjacent hip joint on the left side, **1.2.** PET positive lymph nodes on [^18^F]F-FAPI- 74 PET/CT, The tumor in **A** (H&E) shows a marked but patchy desmoplastic reaction with strong stromal FAP expression in immunohistochemistry (**B**, FAP-ICH).(internal iliac right, SUV_max_ 15, SUV_mean_ 11, SAD 1.9 cm). In the right internal iliac space FAPI PET/CT shows a lymph node with high FAP expression (arrow). Next to it physiologic exrection of the tracer in the bladder, **1.3.** PET negative lymph nodes in [^18^F]F-FAPI- 74 PET/CT, In **A** (H&E) cancer cells showed strong FAP positivity, while the adjacent stroma with dense infiltration by inflammatory cells showed only single-cell FAP expression (**B**, FAP-IHC) (pelvic left, SUV_max_ 3.9, SUV_mean_ 3.4, SAD 1.0 cm). FAPI PET/CT shows only slight, non-suspicious FAP expression in the lymph node in the left pelvic region (arrow). Next to it physiologic exrection of the tracer in the ureter. **1.4.** PET negative lymph nodes on [^68^Ga]Ga-FAPI- 46 PET/CT, The desmoplastic stromal reaction in **A** (H&E) consists mainly of thin collagenous septa surrounding tumor nests. Moderate immunoreactivity for FAP in stromal cells is seen in immunohistochemistry (**B**, FAP-IHC) (pelvic right, SUV_max_ 2.6, SUV_mean_ 2.3, SAD 0.9 cm). FAPI PET/CT shows no relevant FAP expression in the lymph node in the right pelvic region (arrow). Next to it physiologic excrection of the radiotracer in the ureter, same on the left side

Histopathologically positive lymph nodes were associated with an overall significantly higher mean SUV_max_ (7.7 ± 8.8 vs. 1.8 ± 4.0; *p* < 0.001), mean SUV_mean_ (5.3 ± 4.6 vs. 1.4 ± 2.9: *p* < 0.0001) and mean SUV_max_lymph node_/SUV_mean___background_ ratio (16.1 ± 16.8 vs. 3.5 ± 6.7; *p* < 0.0001), for [^18^F]F-FAPI- 74 mean SUV_max_ (6.2 ± 4.4 vs 1.4 ± 0.9; *p* < 0.0001), mean SUV_mean_ (4.5 ± 3.3 vs. 1.1 ± 0.5; p < 0.0001), mean SUV_max_lymph node_/SUV_mean___background_ ratio (10.5 ± 7.2 vs. 2.5 ± 1.5; *p* < 0.0001) and for [^68^Ga]Ga-FAPI- 46 mean SUV_max_ (8.9 ± 11.4 vs 2.4 ± 6.2; *p* = 0.0004), mean SUV_mean_ (6.0 ± 5.5 vs. 1.8 ± 4.5; *p* = 0.0005), and mean SUV_max_lymph node_/SUV_mean___background_ ratio (20.6 ± 21.1 vs. 5.0 ± 10.4; *p* = 0.001) and a greater mean SAD (0.9 ± 0.3 cm vs. 0.6 ± 0.2 cm; *p* < 0.0001) (Fig. 1) than histopathologically negative lymph nodes [^18^F]F-FAPI- 74 1.1 ± 0.3 cm vs 0.6 ± 0.2 cm and for [^68^Ga]Ga-FAPI- 46 0.8 ± 0.2 cm vs 0.6 ± 0.2 cm) (Fig. 1).

### Correlation of lymph node size and FAPI uptake

Overall, mean SUV_max_, mean SUV_mean_ and mean SUV_max_lymph node_/SUV_mean___background_ ratio from both tracers showed a significant correlation with overall mean SAD, for mean SUV_max_ r = 0.52, (95% CI, 0.350–0.670), *p* < 0.01, for mean SUV_mean_ r = 0.49, (95% CI, 0.309–0.644), *p* < 0.01 and for mean SUV_max_lymph node_/SUV_mean___background_ ratio r = 0.57, (95% CI, 0.408–0.706), *p* < 0.001.

Histopathologically positive lymph nodes also revealed a significant correlation between FAPI uptake and SAD, for mean SUV_max_, mean SUV_mean_ and mean SUV_max_lymph node_/SUV_mean___background_ ratio, r = 0.64, (95% CI, 0.264–0.848), *p* = 0.002; r = 0.60, (95% CI, 0.210–0.831), *p* = 0.005 and r = 0.52, (95% CI, 0.092–0.789), *p* = 0.02. Histopathologically negative lymph nodes showed a less significant correlation, for mean SUV_max_ r = 0.27, (95% CI, 0.023–0.490), *p* = 0.028, and for mean SUV_max_lymph node_/SUV_mean___background_ ratio r = 0.36, (95% CI, 0.129–0.566), *p* = 0.003 and no significant correlation for mean SUV_mean_ r = 0.20, (95% CI, − 0.049–0.432), *p* = 0.103.

[^18^F]F-FAPI- 74 revealed a significant correlation regarding FAPI uptake and SAD of lymph node metastases, for mean SUV_max_ r = 0.83, (95% CI, 0.388–0.964), *p* = 0.005, for mean SUV_mean_ r = 0.90, (95% CI, 0.600–0.979), *p* < 0.001, for mean SUV_max_lymph node_/SUV_mean___background_ r = 0.84, (95% CI, 0.415–0.966), *p* = 0.004. Histopathologically negative lymph nodes also showed a less significant uptake/SAD correlation, for mean SUV_max_ r = 0.34, (95% CI, 0.024–0.602), *p* = 0.031, and no significant correlation for mean SUV_mean_, r = 0.29, (95% CI,—0.035–0.563), *p* = 0.071 and mean SUV_max_lymph node_/SUV_mean___background_ ratio r = 0.27, (95% CI,—0.057–0.548), *p* = 0.094.

[^68^Ga]Ga-FAPI- 46 showed a significant FAPI uptake/SAD correlation in lymph node metastases, for mean SUV_max_ r = 0.67, (95% CI, 0.105–0.910), *p* = 0.027, and for mean SUV_mean_ r = 0.68, (95% CI, 0.127–0.914) *p* = 0.024, but a borderline, nonsignificant correlation for mean SUV_max_lymph node_/SUV_mean___background_ ratio r = 0.60, (95% CI,—0.012–0.888) *p* = 0.053. Histopathologically negative lymph nodes showed no significant uptake/SAD correlation, for mean SUV_max_ r = 0.21, (95% CI,—0.202–0.562), *p* = 0.298, for mean SUV_mean_, r = 0.08, (95% CI,—0.320–0.469), *p* = 0.667 and for mean SUV_max_lymph node_/SUV_mean___background_ ratio r = 0.38, (95% CI,—0.014–0.679), *p* = 0.052.

### Biodistribution

Overall mean SUV_mean_ FAPI uptake in liver, blood pool, renal cortex and mesenterial/retroperitoneal fat was 1.2 ± 0.5, 2.2 ± 1.0, 3.0 ± 1.2 and 0.5 ± 0.2 respectively. [^18^F]F-FAPI- 74 revealed a significantly greater FAPI uptake than [^68^Ga]Ga-FAPI- 46 in the liver (1.4 ± 0.4 vs. 0.9 ± 0.5; *p* < 0.0001), blood pool (2.7 ± 0.7 vs. 1.4 ± 0.7; *p* < 0.0001), renal cortex (3.3 ± 1.1 vs. 2.5 ± 1.2; *p* < 0.0001) and mesenterial/retroperitoneal fat (0.6 ± 0.2 vs. 0.4 ± 0.1; *p* < 0,0001).

### Diagnostic performance by visual analysis

Overall sensitivity and specificity for both FAPI-radiotracers were 60.0% and 97.0%. Positive predictive value (PPV) and negative predictive value (NPV) were 80.0% and 92.5%. CT analysis of locoregional lymph nodes showed a sensitivity, specificity, PPV and NPV of 30.0%, 97.0%, 66.6% and 87.7%, respectively. For [^68^Ga]Ga-FAPI- 46 and [^18^F]F-FAPI- 74 sensitivity, specificity, PPV and NPV were 63.6%, 95.8%, 77.7%, 92.0% and 55.5%, 98.1%, 83.3% and 93.1%, respectively (Table 1).

**Table 1:** Visual analysis: consensus read of diagnostic performance comparing [^18^F]F-FAPI- 74 and [^68^Ga]Ga-FAPI- 46

	FAPI overall		[^18^F]F-FAPI- 74		[^68^Ga]Ga-FAPI- 46	
	posLN (*n* = 20)	negLN (*n* = 103)	posLN (*n* = 9)	negLN (*n* = 55)	posLN (*n* = 11)	negLN (*n* = 48)
PET/CT Read						
PET positive	12	3	5	1	7	2
PET negative	8	100	4	54	4	46
CT Read						
CT positive	6	3	4	2	2	1
CT negative	14	100	5	53	9	47

posLN: lymph node region with histopathologically proven lymph node metastases

negLN: lymph node region with histopathologically proven benign lymph node

PET positive: lymph node region with visually assessed markedly elevated FAP expression

PET negative: lymph node region without visually assessed elevated FAP expression

CT positive: lymph node region with visually assessed threshold SAD > 1.0 cm or suspect morphology

CT negative: lymph node region without visually assessed threshold SAD > 1.0 cm or suspect morphology

### Quantitative analysis: ROC analysis

For [^68^Ga]Ga-FAPI- 46, ROC analysis showed an area under the curve for SUV_max_ of 0.85 (95% CI, 0.725–0.980), *p* < 0.001, for SUV_mean_ of 0.84 (95% CI, 0.706- 0.989), *p* = 0.0009, for SUV_max_lymph node)_/SUV_mean___background_ ratio of 0.83 (95% CI, 0.688- 0.976), *p* = 0.0016 to discriminate between histopathologically proven and non-proven lymph node metastases with an optimal cut-off of SUV_max_ at 1.35 resulting in a sensitivity and specificity of 81.8%/89.5% and PPV and NPV of 64.2%/95.5%, for SUV_mean_ with an optimal cut-off at 1.20 resulting in a sensitivity and specificity of 81.8%/89.5% and PPV and NPV of 64.2%/95.5, for SUV_max_lymph node)_/SUV_mean___background_ ratio with an optimal cut-off at 5.95 resulting in a sensitivity and specificity of 72.7%/93.7% and PPV and NPV of 72.7%/93.7% (Table 2).

**Table 2:** Quantitative analysis: ROC analysis of diagnostic performance

ROC analyis of [^68^Ga]Ga-FAPI- 46 diagnostic performance using SUV-based cutoffs		
[^68^Ga]Ga-FAPI- 46		
Cut off value (SUV_max_)	posLN (*n* = 11)	negLN (*n* = 48)
> 1.35	9	5
< 1.35	2	43
Cut off value (SUV_mean_)		
> 1.20	9	5
< 1.20	2	43
Cut off value (SUV_max_/SUV_mean_)		
> 5.95	8	3
< 5.95	3	45
ROC analyis of [^18^F]F-FAPI- 74 diagnostic performance using SUV-based cutoffs		
[^18^F]F-FAPI- 74		
Cut off value (SUV_max_)	posLN (*n* = 9)	negLN (*n* = 55)
> 1.55	9	10
< 1.55	0	45
Cut off value (SUV_mean_)		
> 1.25	9	13
< 1.25	0	42
Cut off value (SUV_max_/SUV_mean_)		
> 4.15	8	3
< 4.15	1	52
ROC analyis of SAD (both tracer groups) diagnostic performance using SAD cutoff		
SAD overall		
Cut off value (cm)	posLN (*n* = 20)	negLN (*n* = 103)
> 0.85	15	16
< 0.85	5	87

posLN: lymph node region with histopathologically proven lymph node metastases

negLN: lymph node region with histopathologically proven benign lymph node

SAD: short axis diameter

For [^18^F]F-FAPI- 74, ROC-analysis showed an area under the curve for SUV_max_ of 0.92 (95% CI, 0.839–1.000, *p* < 0.001, for SUV_mean_ of 0.91 (95% CI, 0.830—0.1000), *p* = 0.0001, for SUV_max_lymph node_/SUV_mean___background_ ratio of 0.97 (95% CI, 0.927- 1.000), *p* < 0.0001 to discriminate between histopathologically proven and non-proven lymph node metastases with an optimal cut-off off SUV_max_ at 1.55 which results in a sensitivity and specificity of 100%/81.8% and PPV and NPV of 47.3%/100%, for SUV_mean_ with an optimal cut-off at 1.25 resulting in a sensitivity and specificity of 100%/93.3% and PPV and NPV of 40.9%/100%, for SUV_max_lymph node_/SUV_mean___background_ ratio with an optimal cut-off at 4.15 resulting in a sensitivity and specificity of 88.8%/9 4.5% and PPV and NPV of 72.7%/98.1% (Table 2).

ROC-analysis of overall SAD revealed an area under the curve of 0.80 (95% CI, 0.668—0.933), *p* < 0.001 to discriminate between histopathologically proven and non-proven lymph node metastases with an optimal cut-off off 0.85 cm which results in a sensitivity and specificity of 75.0%/84.4% and PPV and NPV of 48.3%/94.5% (Table 2).

Comparing the visual assessment with the quantitative analysis revealed a lower sensitivity in the visual read compared to the quantitative analysis ([^68^Ga]Ga-FAPI- 46 63.6% vs. SUV_max_ 81.8%, SUV_mean_ 81.8%, SUV_max_lymph node_/SUV_mean___background_ ratio 72.7%; [^18^F]F-FAPI- 74 55.5% vs. SUV_max_ 100%, SUV_mean_ 100%, SUV_max_lymph node_/SUV_mean___background_ ratio 88.8%) whereas specificity showed higher values in the visual assessment than in the quantitative analysis ([^68^Ga]Ga-FAPI- 46 95.8% vs. SUV_max_ 89.5%, SUV_mean_ 89.5%, SUV_max_lymph node_/SUV_mean___background_ ratio 93.7%; [^18^F]F-FAPI- 74 98.1% vs. SUV_max_ 81.8%, SUV_mean_ 93.3%, SUV_max_lymph node_/SUV_mean___background_ ratio 94.5%) (Table 3).

**Table 3:** Comparison of visual and quantitative analysis of diagnostic performance

	[^68^Ga]Ga-FAPI- 46	[^18^F]F-FAPI- 74	SAD both tracers
Sensitivity	Visual Read: 63.6% Quantitative Analysis SUVmax: 81.8% Quantitative Analysis SUVmean: 81.8% Quantitative Analysis SUVratio: 72.7%	Visual Read: 55.5% Quantitative Analysis SUVmax: 100.0% Quantitative Analysis SUVmean: 100. 0% Quantitative Analysis SUVratio: 88.8%	Visual Read: 30.0% Quantitative Analysis: 75.0%
Specificity	Visual Read: 95.8% Quantitative Analysis SUVmax: 89.5% Quantitative Analysis SUVmean: 89.5% Quantitative Analysis SUVratio: 93.7%	Visual Read: 98.1% Quantitative Analysis SUVmax: 81.8% Quantitative Analysis SUVmean: 93.3% Quantitative Analysis SUVratio: 94.5%	Visual Read: 97.0% Quantitative Analysis: 84.4%
PPV	Visual Read: 77.7% Quantitative Analysis SUVmax: 64.2% Quantitative Analysis SUVmean: 64.2% Quantitative Analysis SUVratio: 72.7%	Visual Read: 83.3% Quantitative Analysis SUVmax: 47.3% Quantitative Analysis SUVmean: 40.9% Quantitative Analysis SUVratio: 72.7%	Visual Read: 66.6% Quantitative Analysis: 48.3%
NPV	Visual Read: 92.0% Quantitative Analysis SUVmax: 95.5% Quantitative Analysis SUVmean: 95.5% Quantitative Analysis SUVratio: 93.7%	Visual Read: 93.1% Quantitative Analysis SUVmax: 100.0% Quantitative Analysis SUVmean: 100.0% Quantitative Analysis SUVratio: 98.1%	Visual Read: 87.7% Quantitative Analysis: 94.5%

SUVratio: ratio between SUVmax of lymph node and SUVmean of mesenterial/retroperitoneal fat: SUV_max_lymph node_/SUV_mean___background_ *SAD* short axis diameter

ROC-analysis for overall lymph node SAD at a cutoff of 0.8 cm would result in a higher sensitivity than the visual analysis of both tracers (75.0% v.s 63.6% [^68^Ga]Ga-FAPI- 46 and 55.5% [^18^F]F-FAPI- 74) as well as in a lower specificity (84.4% v.s 95.8% [^68^Ga]Ga-FAPI- 46 and 98.1% [^18^F]F-FAPI- 74) (Table 3). Compared to the ROC-analyses of both tracers, SUV-based assessment would outperfom SAD analysis most of the times (Table 3).

### Lymph nodes: False-positive/false-negative findings

There were 8/123 (6.5%) histopathologically proven lymph node metastasis regions in 7/43 patients without any significantly increased FAPI uptake in at least one lymph node ([^18^F]F-FAPI- 74: *n* = 4; [^68^Ga]Ga-FAPI- 46 *n* = 4). An increased FAPI uptake was seen in three lymph node regions, including one region in [^18^F]F-FAPI- 74 and two regions in [^68^Ga]Ga-FAPI- 46 without histopathologically confirmed lymph node metastases. These regions contained two CT positive lymph nodes (SAD 1.4 cm and 1.3 cm) while the third lymph node was not pathologically enlarged (SAD 0.6 cm).

### Primary tumor

**Fig. 2:**
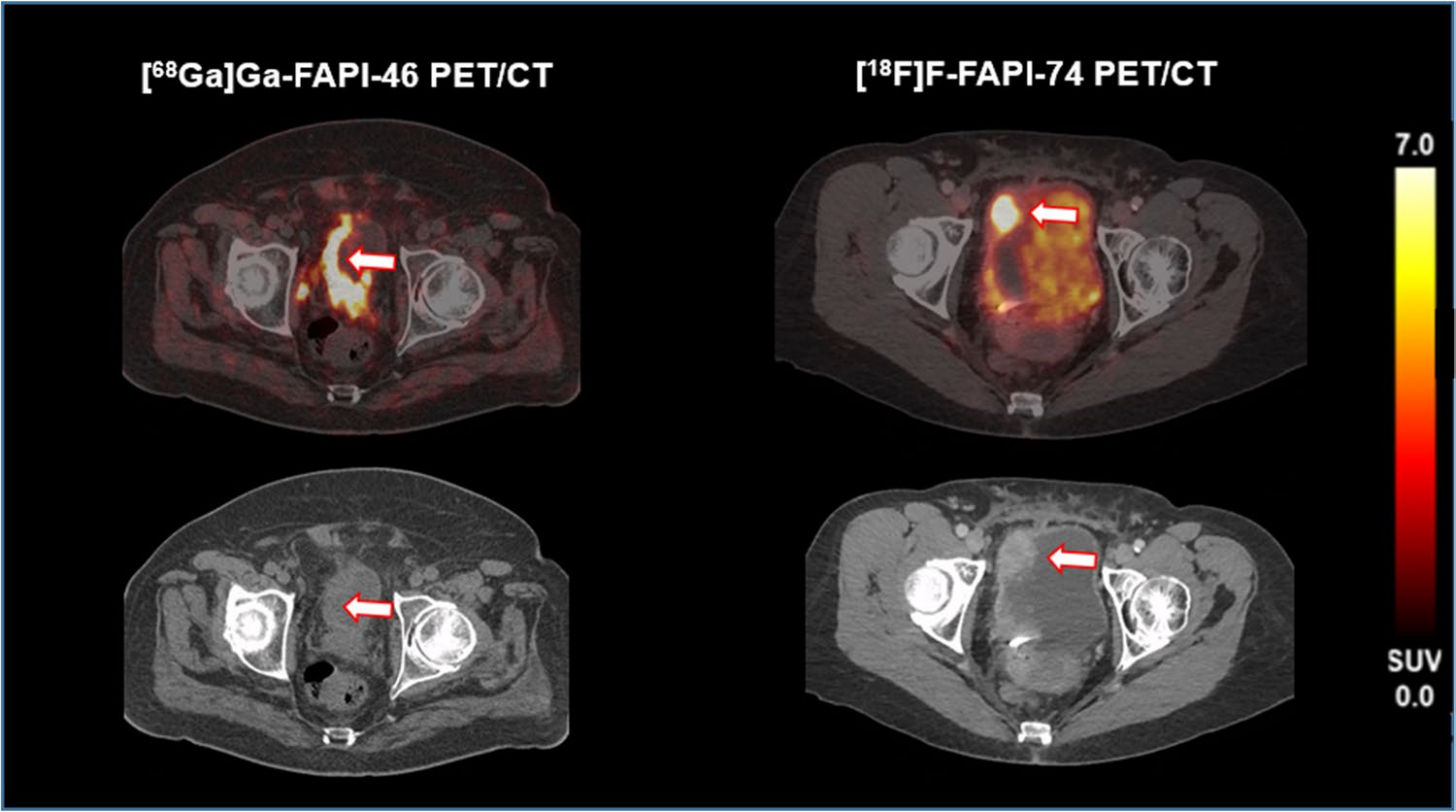
Increased FAPI uptake in the primary tumor in both tracers, Left: [^68^Ga]Ga-FAPI- 46 PET/CT FAP expressing primary (SUV_max_ 21, SUV_mean_ 16); Right: [^18^F]F-FAPI- 74 PET/CT FAP expressing primary (SUV_max_ 24, SUV_mean_ 19). Low dose CT on the left shows an asymmetric thickening of the bladder (arrow), contrast enhanced CT on the right side shows focal tumor tissue (arrow), both findings corresponding with the primary

In 13 of 51 (25.4%) patients, the primary tumor in the bladder showed an increased FAPI uptake (SUV_max_ 17.1 ± 7.0, SUV_mean_ 14.5 ± 6.6; [^18^F]F-FAPI- 74: 8/28, 28.5%, SUV_max_ 16.1 ± 8.6, SUV_mean_ 12.9 ± 7.9; [^68^Ga]Ga-FAPI- 46; 5/23, 21.7%, SUV_max_ 18.8 ± 1.4, SUV_mean_ 17.1 ± 2.8). There was no significant difference in the SUV_max_ of both groups (16.3 vs 18.8; *p* = 0.22) and SUV_mean_ (12.9 vs. 17.1; *p* = 0.17). Visual assessment showed no relevant differences between the FAPI uptake of both tracers (Fig. 2). Three patients ([^18^F]F-FAPI- 74 *n* = 2, [^68^Ga]Ga-FAPI- 46 *n* = 1) showed a CT morphologically visible primary without increased FAPI uptake as far as distinguishable from the surrounding high urine activity of both tracers.

### Distant metastases

**Fig. 3:**
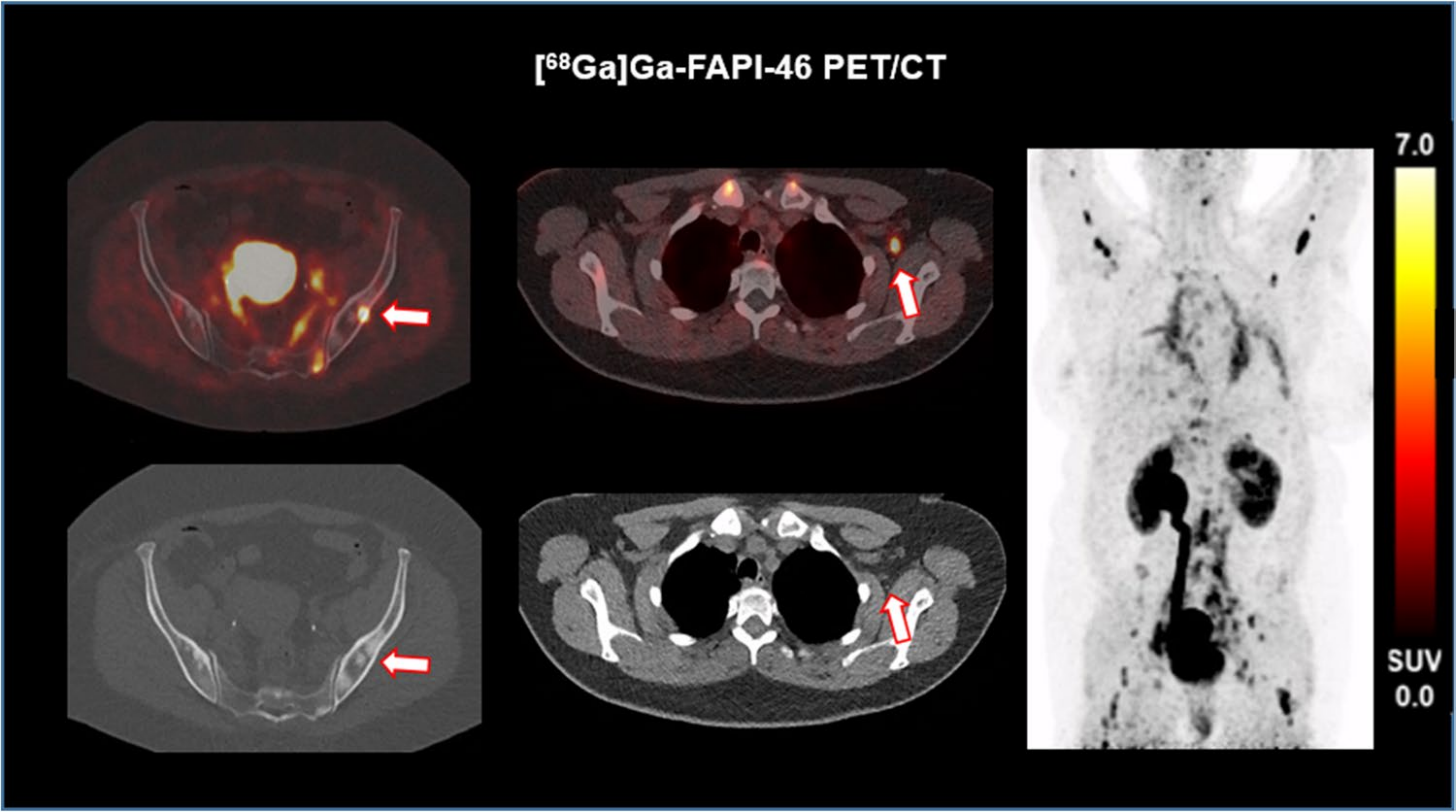
Increased [^68^Ga]Ga-FAPI- 46 expression in a patients with multiple bone and lymph node metastases who underwent radical cystectomy due to non-stoppable hematuria, Left: bone metastasis in the left ileum bone (SUVmax. 8.5, SUV_mean_ 7.9) (arrow); Right: left axillary lymph node metastasis (SUVmax. 9.9, SUV_mean_ 7.4) (arrow). The patient revealed an extensive nodal spread to mediastinum, left axilla, paraaortic and pelvic, pulmonary metastases and disseminated metastatic osseous spread

Three patients presented with distant (lymph node metastases *n* = 3 (affection of paraaortal and mediastinal lymph node metastases), peritoneal metastases *n* = 2, bone metastases *n* = 1), FAP positive metastases at the preoperative FAPI scan (all [^68^Ga]Ga-FAPI- 46 scans). These patients had recurrent non-stoppable macrohematuria (1 patient under oral anticoagulation for concomitant atrial fibrillation). Overall, those metastases showed a markedly elevated FAPI uptake (SUV_max_ 7.9 ± 6.8, SUV_mean_ 6.4 ± 4.2) (Fig. 3).

## Discussion

The central task of preoperative tumor diagnostics in muscle-invasive BC is the accurate identification of local or distant metastases, as the nodal status fundamentally impacts treatment and prognosis. For instance, systemic (palliative) therapy is recommended in patients with nodal spread, whereas neoadjuvant therapy and RC should be performed in node negative patients. Conventional imaging with contrast-enhanced CT is recommended by current guidelines for baseline and follow-up staging, but its diagnostic accuracy in the detection of lymph node metastases is low [1, 2, 8–12]. In order to improve diagnostic performance, [^18^F]FDG PET/CT has already been evaluated, but could only show a small benefit and is not included in standard clinical workup protocols [1, 2]. Therefore, the establishment of novel imaging approaches is crucial for treatment planning and improving oncological outcome in patients.

In a pilot cohort we showed that hybrid imaging using [^68^Ga]Ga-FAPI- 46 PET/CT offers a higher sensitivity in detecting lymph node metastasis compared to conventional imaging alone [17].

Recently, there has been an increased interest in comparing ^18^F labeled FAPI tracers to ^68^ Ga labeled tracers and [^18^F]FDG. [^18^F]F-FAPI tracers have already shown good intra-tumoral accumulations in tumor entities such as non-small cell lung cancer [21] as well as encouraging results for detection rates in comparison to [^18^F]FDG in pancreatic cancer [23]. ^18^F labeled tracers possess an improved spatial resolution due to lower positron energy [24] and are more suited to meet the increasing demand for reliable diagnostic tracers. In contrast, ^68^ Ga labeled tracers require multiple syntheses for large center supplies and have limitations in long-distance shipping due to their shorter half-life [25]. In addition, reduced urinary excretion of [^18^F]F-FAPI- 74 could allow for earlier detection of smaller lesions in the urinary tract. Thus, in this study, we aimed to assess the potential benefits of [^18^F]F-FAPI- 74 compared to ^68^ Ga-FAPI in detecting lymph node metastases in BC patients before RC.

Within this study, we reproduced our previous findings regarding the performance of [^68^Ga]Ga-FAPI- 46 in detecting local lymph node metastases [17]. Addressing [^18^F]F-FAPI- 74, this tracer shows similar sensitivity and specificity compared to [^68^Ga]Ga-FAPI- 46. Lymph node metastases showed significantly higher SUV_max_ and SUV_mean_ values as well as SUV_max_lymph node_/SUV_mean___background_ ratios (SUV_max_ lymph node/SUV_mean_ of mesenterial/retroperitoneal fat) than histopathologically negative lymph nodes (Fig. 1). In addition to that, both tracers showed a significant correlation between FAPI uptake and SAD in lymph node metastases while histopathologically unremarkable lymph nodes revealed a mostly less or not significant correlation.

Concordant with our previous findings, both radiotracers proved to have a superior detection of lymph node metastases in comparison to conventional CT.

Comparing results of the visual and quantitative analysis, ROC analysis revealed an overall higher sensitivity but lower specificity for both, [^68^Ga]Ga-FAPI- 46 and [^18^F]F-FAPI- 74. This could be due to the fact, that the FAPI uptake based-cutoffs calculated by the quantitative analysis were markedly lower, increasing the detection rate of FAPI uptake while also raising the probability of false positive findings, thus lowering the specificity. It is worth noting, that the number of included lymph nodes included in the ROC analysis was quite low, which may have affected the calculation of quantitative values. This would also explain similar values for diagnostic performances between SUV_max_ and SUV_mean_ as the calculated cutoffs are very close as seen in the SUV_max_ and SUV_mean_ ROC analysis of [^68^Ga]Ga-FAPI- 46. This issue should be addressed in larger cohorts.

Interpreting our three SUV based methods, in the [^68^Ga]Ga-FAPI- 46 cohort, SUV_max_ and SUV_mean_ values seem to have the best diagnostic performance while SUV_mean_ values revealed slightly higher specifity in the [^18^F]F-FAPI- 74 subgroup. [^68^ Ga]Ga-FAPI- 46 SUV_max_lymph node_/SUV_mean___background_ ratios show a lower sensitivity than the rest of the SUV values while displaying excellent specifity in both tracer subcohorts.

Overall, there is no single parameter,that shows superior sensitivity and specifity combined in contrast to the other parameters. Thus we refrain from proposing a defintive cutoff based on a single SUVbased assessment especially as this is a small cohort and a retrospective imaging analysis.

In addition to the above SUV-based tracers, also a SAD-based cut-off should be evaluated in a a prospective study design: Histopathologically positive lymph nodes showed significant higher SAD with a mean SAD of 0.8 cm for [^68^Ga]Ga-FAPI- 46 and 1.1 cm for [^18^F]F-FAPI- 74. Additionally, our ROC analysis of overall SAD proposed a cutoff of 0.8 cm with an acceptable diagnostic performance (Table 3). These findings are in line with current guidelines, where in conventional imaging pelvic lymph nodes with an SAD > 0.8 cm should be considered “as pathologically enlarged” [1].

Both tracer groups contained patients with markedly FAPI positive lymph nodes without the presence of metastases. As we previously hypothesized, this could be due to non-malignant processes such as inflammatory reactions or the presence of non-resected metastases [17]. Interestingly, one FAP negative lymph node exhibited an increased FAP expression by the cancer cells themselves, while the tumor-associated stroma showed only minor FAPI uptake (Fig. 1).

Most of our study population did not show visually elevated FAP overexpression in the primary bladder tumor. This is primarily caused by the urinary excretion of the tracer, but may also be attributed to the extensive pretreatment with transurethral resection in the biggest portion of patients, or the possibility of FAP negative carcinomas, as three patients presented with a morphologically visible primary tumor without FAPI uptake. In cases of FAPI positive primaries, both tracers showed comparable SUV_max_ and SUV_mean_ values. Although we hypothesized, that [^18^F]F-FAPI- 74 would be superior in detecting intravesical lesions due to the reduced renal excretion and therefore improve tumor to background ratios [17], this was not observed in our current pilot study; here, further investigations are needed. The observed biodistribution of [^18^F]F-FAPI- 74 seems in line with previous findings [16, 26].

Given the similar performance of both tracers, [^18^F]F-FAPI- 74 has the potential to offer a significant benefit for clinical practice as a staging tool in patients with advanced and metastatic disease. In our previous study, we demonstrated good tracer uptake in distant metastases, where CT scans had indicated unremarkable findings. Of note, morphologically suspicious imaging findings without FAPI expression were confirmed as benign by histopathology [18]. Even in small lesions FAPI PET/CT displayed superior diagnostic performance over conventional imaging [27].

In addition to the visual assessment, our study demonstrated an excellent diagnostic performance using predefined SUV-based cutoffs for FAPI PET/CT, suggesting that this could provide a simple and widely accessible screening method for lymph node status prior to RC.

Since FAPI PET/CT is not as available as conventional imaging outside of academic settings, further studies need to evaluate two decisive factors: First, an optimal threshold for the lymph node size at which FAPI PET/CT provides the greatest benefit should be evaluated to refer patients at risk to further imaging. Secondly, the conditions under which CT-guided biopsy should be considered in inconclusive cases in order to confirm metastatic lymph nodes and overall improve oncological outcome. Two workup routines can be discussed for the second scenario evaluating inconclusive findings on conventional images.

Regarding our findings, we suggest, that FAPI PET/CT should be considered in all inconclusive findings in conventional imaging suspecting lymph node metastases with SAD > 0.8 cm and/or lymph nodes showing suspicious morphology or localization. If false positive findings are suspected, CT-guided biopsy should be indicated as unnecessary metastatic resection can be associated with risk of local complications as bleeding, infection and nerve damage or an initial curative approach would be wrongfully dismissed in the presence of alleged distant metastases. FAPI PET/CT could also serve to examine false positive findings on conventional imaging to avoid the need for further biopsy and its associated complications.

False negative findings would pose the greatest risk for insufficient therapy as FAPI PET/CT has already revealed superior diagnostic performance compared to using established RECIST criteria in conventional imaging in urothelial carcinoma [27], thus limiting imaging options. Next to histopathologic workup via biopsy, if FAPI negative lesions are suspected, one might consider a different malignancy with, depending on subtype, varying and/or partially low FAP expression like lymphoma [28]. Additional hybrid imaging, using FDG PET/CT could evaluate those inconclusive lesions.

Our study has several limitations: Due to its retrospective design patients did not follow a standardized protocol for PLND, which might have affected adequate removal of lymph node metastases. A prospective approach could improve lymph node mapping and allow for comparisons between different extents of PLND and outcome. Additionally, we performed an interindividual comparison of patients receiving PET/CT, therefore individual uptake values can vary depending on different pretreatments. Further studies focusing on an intraindividual comparisons are needed to eliminate patient related influences. Due to the limited patient population, several analyses such as the comparison of the neoadjuvant chemotherapy subgroup to the main population could not be performed, thus this needs to be addressed in following studies.

## Supplementary Information

Below is the link to the electronic supplementary material.

Supplementary file1 (DOCX 18 KB)

## Data Availability

The datasets generated during and/or analysed during the current study are available from the corresponding author on reasonable request.

## Declarations

### Precursors

The precursors for both used radiotracers were provided by SOFIE (21000 Atlantic Blvd., Ste 730, Dulles, VA 20166).

### Clinical trial number

Clinical trial number: not applicable.

## Abbreviations

*BC*: Bladder cancer
*CT*: Computed tomography
*CAF*: Cancer-activated fibroblast
*FAPI*: Fibroblast activation protein inhibitor
*FAP*: Fibroblast activation protein
*FDG*: 2-[^18^F]fluoro- 2-deoxy-D-glucose
*GMP*: Good manufacturing practice
*kV*: KiloVolt
*mAs*: Milliampere-seconds
*MeV*: Megaelectron volt
*MIBC*: Muscle invasive bladder cancer
*MRI*: Magnetic resonance imaging
*NPV*: Negative predictive value
*PET*: Positron emission tomography
*PLND*: Pelvic lymph node dissection
*PPV*: Positive predictive value
*PSMA*: Prostataspecific membrane antigen
*RC*: Radical cystectomy
*SAD*: Short axis diameter
*SUV*_*max*_: Maximum standardized uptake value
*SUV*_mean_: Mean standardized uptake value
*VOI*: Volume of interest
