# Supplementary material for: 68Ga-FAPI and 18F-FAPI PET/CT for detection of nodal metastases prior radical cystectomy in high-risk urothelial carcinoma patients
Source: Eur J Nucl Med Mol Imaging. 2025 Apr 24;52(11):3963–74. doi: 10.1007/s00259-025-07239-6 (PMC12397140; doi:10.1007/s00259-025-07239-6)
Supplement: Supplementary file 1 — Supplementary file1 (DOCX 18 KB) [file 259_2025_7239_MOESM1_ESM.docx]

**Supplementary information for study “68Ga-FAPI and 18F-FAPI PET/CT for detection of nodal metastases prior radical cystectomy in high-risk urothelial carcinoma patients” EJNMMI: Baseline patient characteristics**

| No | Age (years) | Sex | Localization | Radical local therapy prior to PET | Systemic therapy prior to PET | Highest pT stage | Tumor grade | Metastastic disease | Localization of metastases | FAP positive Local tumor burden/distinguishable from urinary activity |
| --- | --- | --- | --- | --- | --- | --- | --- | --- | --- | --- |
| 1 | 81 | M | Bladder | Y | N | 4a | 3 | N | - | - |
| 2 | 87 | M | Bladder | Y | N | 3a | High grade | N | - | - |
| 3 | 63 | M | Bladder | y | N | 2a | 3 | N | - | - |
| 4 | 82 | M | Bladder | Y | N | 2b | High grade | N | - | - |
| 5 | 60 | M | Bladder | Y | Y | 2a | 2 | N | - | - |
| 6 | 78 | M | Bladder | Y | N | 3a | 3 | N | - | - |
| 7 | 81 | M | Bladder | Y | N | 3b | 3 | Y | Locoregional | Y |
| 8 | 56 | M | Bladder | Y | N | 4a | 3 | N | - | - |
| 9 | 63 | M | Bladder | Y | N | 3a | High grade | N | - | - |
| 10 | 76 | M | Bladder | Y | N | a | 3 | N | - | Y |
| 11 | 75 | M | Bladder | Y | Y | 1 | 3 | N | - | - |
| 12 | 65 | M | Bladder | Y | Y | 2a | 3 | N | - | - |
| 13 | 83 | M | Bladder | Y | N | 4a | High grade | Y | Locoregional and distant | Y |
| 14 | 77 | M | Bladder | y | N | 2b | 3 | Y | Locoregional | - |
| 15 | 55 | M | Bladder | Y | N | 1a | 3 | N | - | - |
| 16 | 82 | M | Bladder | Y | N | 2a | 3 | N | - | - |
| 17 | 80 | M | Bladder | Y | N | 2b | 3 | Y | Locoregional and distant | - |
| 18 | 84 | M | Bladder | Y | N | a | High grade | N | - | - |
| 19 | 74 | M | Bladder | Y | N | 3b | 3 | Y | Locoregional | - |
| 20 | 72 | M | Bladder | Y | Y | 3a | 3 | N | - | - |
| 21 | 75 | M | Bladder | Y | N | 1 | 3 | N | - | - |
| 22 | 79 | M | Bladder | Y | N | 2a | 3 | N | - | - |
| 23 | 66 | M | Bladder | Y | N | 2a | 3 | Y | Locoregional | - |
| 24 | 71 | M | Bladder | Y | Y | 1 | High grade | N | - | - |
| 25 | 58 | M | Bladder | Y | N | 3a | 3 | Y | Locoregional | Y |
| 26 | 66 | M | Bladder | Y | N | 2a | 3 | N | - | - |
| 27 | 60 | M | Bladder | Y | N | 2a | 3 | N | - | - |
| 28 | 62 | M | Bladder | Y | N | 1 | 3 | N | - | - |
| 29 | 74 | M | Bladder | Y | Y | 2 | 3 | N | - | - |
| 30 | 87 | M | Bladder | Y | N | 2 | 3 | N | - | - |
| 31 | 82 | M | Bladder | Y | N | 2b | 3 | N | - | - |
| 32 | 74 | M | Bladder | Y | N | 1 | 3 | N | - | - |
| 33 | 72 | W | Bladder | Y | N | 4a | 3 | Y | Locoregional | Y |
| 34 | 64 | M | Bladder | Y | N | 4a | High grade | N | - | - |
| 35 | 78 | M | Bladder | Y | N | 3a | 3 | Y | Locoregional | - |
| 36 | 66 | W | Bladder | Y | N | 4a | 3 | Y | Locoregional and distant | Y |
| 37 | 63 | M | Bladder | Y | Y | 1 | 3 | N | - | - |
| 38 | 70 | M | Bladder | Y | N | 2 | 3 | N | - | - |
| 39 | 74 | M | Bladder | Y | N | 2b | 3 | Y | - | - |
| 40 | 67 | M | Bladder | Y | N | 2b | 3 | N | - | - |
| 41 | 58 | W | Bladder | Y | Y | 4b | 3 | Y | Locoregional | Y |
| 42 | 74 | W | Bladder | Y | N | 2a | 3 | N | - | - |
| 43 | 74 | M | Bladder | Y | N | 3b | 3 | N | - | - |
| 44 | 69 | W | Bladder | Y | N | 3a | 3 | n/a | - | Y |
| 45 | 89 | M | Bladder | Y | N | 2 | 3 | n/a | - | - |
| 46 | 87 | M | Bladder | Y | N | 4b | 3 | n/a | - | Y |
| 47 | 80 | W | Bladder | Y | N | 3a | 3 | n/a | - | Y |
| 48 | 51 | M | Bladder | Y | Y | 3b | 3 | n/a | - | Y |
| 49 | 81 | M | Bladder | Y | N | 3a | 3 | n/a | - | Y |
| 50 | 76 | M | Bladder | Y | N | 3b | 3 | n/a | - | Y |
| 51 | 80 | M | Bladder | Y | N | 1 | 3 | n/a | - | - |

Y: yes

N: no

n/a: not available

Authors: Lena M. Unterrainer*^1,2,3^, Hans Schmid*^1^, Sophie C. Kunte^1,2^, Adrien Holzgreve^1,3^, Johannes Toms^1^, Paula Menold^4^, Clemens C. Cyran^5^, Alexander Karl^4^, Stephan Tschirdewahn^6^, Stephan T. Ledderose^2,7^, Lennert Eismann^8^, Alexander J. Tamalunas^8^, Maximilian Scheifele^1^, Christian G. Stief^8^, Marcus Unterrainer^1,9^, Jozefina Casuscelli^8^ and Gerald B. Schulz^8^

1. Department of Nuclear Medicine, LMU University Hospital, LMU Munich, Munich, Germany
2. Bavarian Cancer Research Center (BZKF), partner site Munich, Germany
3. Ahmanson Translational Theranostics Division, Department of Molecular and Medical Pharmacology, David Geffen School of Medicine, University of California Los Angeles, Los Angeles, California
4. Department of Urology, Krankenhaus Barmherzige Brüder, Munich, Germany
5. Department of Radiology, LMU University Hospital, LMU Munich, Munich, Germany
6. Department of Urology, Klinikum Kempten, Germany
7. Institute of Pathology, LMU Munich, Munich, Germany
8. Department of Urology, LMU University Hospital, LMU Munich, Munich, Germany.
9. Die RADIOLOGIE, Munich, Germany

* contributed equally

**Correspondence:**

Hans Schmid

Marchioninistrasse 15

81377 Munich, Germany

[Hans.Schmid@med.uni-muenchen.de](mailto:Hans.Schmid@med.uni-muenchen.de)
